# Dietary inflammatory index: a potent association with cardiovascular risk factors among patients candidate for coronary artery bypass grafting (CABG) surgery

**DOI:** 10.1186/s12937-018-0325-2

**Published:** 2018-02-13

**Authors:** Mahdieh Abbasalizad Farhangi, Mahdi Najafi

**Affiliations:** 1 Drug Applied Research Center, Tabriz University of Medical Sciences, Tabriz, Iran; 2 Department of Research, Tehran Heart Center, Tehran University of Medical Sciences, North Karegar Street, Tehran, 1411713138 Iran; 3 Cardiac Outcome Research and Education (CORE), Universal Scientific Education and Research Network (USERN), Tehran, Iran

**Keywords:** Dietary inflammatory index, CABG, Hs-CRP, Cardiovascular disease

## Abstract

**Background:**

Recently, the clinical importance of dietary inflammatory index (DII) in predicting the inflammatory potential of diet and its role in pathogenesis of several chronic disease including some types of cancers, osteoporosis, cardiovascular disease and renal disease has been proposed. However, its association with the disease severity and progression and cardio-metabolic risk factors among patients candidate for coronary artery bypass graft surgery (CABG) has not been evaluated yet. In the current study, the association of DII with cardiovascular risk factors among patients candidate for CABG has been investigated.

**Methods:**

In the current cross-sectional study, 454 patients aged 35-80 years as candidates of CABG and hospitalized in Tehran Heart Center were enrolled. Anthropometric and demographic characteristics were obtained from all of the participants. Dietary intakes were evaluated with a semi-quantitative food-frequency questionnaire (FFQ) and DII was calculated. Biochemical parameters including hemoglobin (Hb) A_1_C, serum lipids, albumin, creatinine and high-sensitive C-reactive protein (hs-CRP), lipoprotein (a), creatinine, blood urea nitrogen (BUN), albumin and hematocrit (HCT) were also assessed by commercial laboratory methods. Left ventricular ejection- fraction, number of diseased vessels, New York Heart Association (NYHA) functional class and the European system for cardiac operative risk evaluation (EuroSCORE) were estimated for clinical assessment. One way analysis of variance and chi square tests were used for comparison of demographic parameters between groups. Beta estimates and 95% confidence intervals (CI) for the association between DII and clinical parameters were estimated using linear regression adjusted for the confounders.

**Results:**

According to our findings, high DII scores were associated with higher age, lower body mass index (BMI), higher prevalence of diabetes and myocardial infarction (MI) and lower educational attainment (*P* < 0.05). Male patients in 4th and 3rd quartile of DII had significantly higher total cholesterol (TC), triglyceride (TG), albumin, creatinine, BUN and hs-CRP concentrations and lower high density lipoprotein cholesterol (HDL) concentrations compared with male patients in lower quartiles (*P* < 0.05). While in female patients, only lipoprotein (a) concentrations and hematocrit (HCT) percentage in the 4th and 2nd quartile were significantly higher than lower quartiles. EuroSCORE was also significantly higher in top quartiles of DII (*P* = 0.006).

**Conclusion:**

As shown in our results, DII was in a positive association with several cardiovascular risk factors. The higher inflammatory potential of diet denoted higher values of serum lipids, CRP and kidney function tests and higher EuroSCORE as a predictor of post-operative mortality. Therefore, DII can be demonstrated as a target of nutritional interventions for ameliorating the CVD risk factors among patients candidate for CABG especially in male patients.

## Background

Cardiovascular disease (CVD) is one of the most common causes of morbidity and mortality in different communities accounting for more than 31% or 17.5 million deaths worldwide; more that 75% of these deaths occur in low and middle income countries [1]. In Iran, it is a leading cause of mortality, morbidity, and disability and accounts for nearly 50% of all deaths per year [2]. Coronary artery bypass grafting (CABG) is the most common type of open-heart surgical interventions for the treatment of patients in the higher stages of coronary artery disease (CAD); it is performed for patients with CAD to improve quality of life and reduce cardiac-related mortality [3]. CABG was introduced in the 1960s with the aim of offering symptomatic relief, improved quality of life, and increased life expectancy to patients with CAD [4]. By the 1970s, CABG was found to increase survival rates in patients with multi-vessel disease and left main disease when compared with medical therapy [5]. The surgery is performed where atherosclerosis of one or more of coronary arteries is severe enough to show at least 50% stenosis of arterial lumen in angiographic image. The number of CABG operations carried out to treat CAD has increased more than fivefold since 1980, and the general trend has been an almost steady rise in the number of operations performed each year [6].

Chronic inflammation is a potential triggering factor in the origin of the cardiovascular disease; stimuli such as over-nutrition and physical inactivity would result in cytokine hyper-secretion [7]. Current evidence supports that inflammation is a major driving force in patients with CAD, underlying the initiation of coronary plaques, their unstable progression, and eventual disruption; numerous studies in this field had been performed and revealed that several potential inflammatory biomarkers including high sensitive C-reactive protein (hs-CRP), interleukin (IL)-6, IL-8 and IL-1β are potent inflammatory mediators in progression of CAD and even they can be used as predictive markers in diagnosis of the severity of disease [8]. Interestingly, these data suggest that the increased risk associated with inflammation may be modified with certain preventive therapies and biomarkers may help to identify the individuals who would benefit most from these interventions; numerous drugs are developed for CAD targeting reducing inflammation as the first step of treatment [9]. Even in patients candidate to CABG, the pre-operative levels of inflammatory markers is associated with poor clinical outcomes and increased risk of low cardiac output syndrome (LCOS), postoperative myocardial infarction (PMI) and in-hospital cardiovascular death following elective CABG [10].

Diet and dietary habits play a crucial role in the pathogenesis of cardiovascular disease and dietary habits are potential determinants of the disease severity. The role of dietary factors and nutritional regimens in the prevention of cardiovascular disease (CVD) and its progression has been extensively studied; numerous reports suggested the role of healthy dietary choices and improved life style with higher physical activity level [11] and higher intakes of healthy foods including fruits and vegetables and dietary antioxidants in prevention and treatment of cardiovascular events [12]; however, there is no study evaluating the association between the pro-inflammatory and anti-inflammatory potential of overall diet in patients candidate for CABG. Pro-inflammatory nature of the cardiovascular disease can be explained by this fact that almost in all of the atherosclerosis processes inflammatory molecules are involved; initiation of the atherosclerosis by numerous triggers including saturated fatty acids, hypertension and obesity stimulates the expression of adhesion molecules like vascular cell adhesion molecule − 1 (VCAM-1) in endothelial cells; VCAM expression initiates by means of oxidized lipids by nuclear factor kappa B, IL-1β and TNF-α [13]. In the other stages, development of fatty streaks and progression to complex plaque involves other inflammatory cytokines include monocyte chemoattractant protein (MCP)-1 and macrophage colony-stimulating factor (M-CSF), IL-1β, IL-1α, IL-6, TNF-α, TNF-β and IL-18 [14, 15].

Dietary inflammatory index (DII) is a literature review-based score that reflects the potential inflammatory effects of the diet. It was first developed by Cavicchia et al. [16] and updated by Shivappa et al. [17]. In developing the DII, nearly 2000 papers were reviewed and scored. The scoring algorithm was based on the inflammatory effects of 45 food parameters including foods, nutrients and other bioactive compounds on some specific inflammatory and anti-inflammatory parameters including CRP, IL-1, IL-4, IL-6, IL-8, IL-10 and TNF-α. Choosing these inflammatory parameters was based on their established importance in inflammation and concomitantly the robustness of the literature concerning them. According to the effects of the food parameter on inflammation, one of three values was assigned to each article; ‘+ 1’ was assigned if the effects was pro-inflammatory (significantly increased IL-β, IL-6, TNF-α or CRP). ‘-1’ was assigned if the effects was anti-inflammatory (significantly reduced IL-β, IL-6, TNF-α or CRP or increased IL-4 or IL-10) and ‘0’ was assigned if no significant effect of food parameter on inflammatory markers was achieved. Thereafter, food parameter-specific raw inflammatory effect scores and food parameter-specific overall inflammatory effects scores were developed [17].

Classifying the individual’s diet according to their inflammatory properties could give a practical application to DII in CABG for clinical and public health by targeting nutrition education, counseling and health promotion activities in patients undergoing CABG and to monitor changes in inflammatory potential of diet over time. It can also be used to predict the post-operative dietary risk assessment to develop dietary interventional strategies for prevention of recurrent cardiac events after CABG [18, 19]. Numerous studies had evaluated the association between DII and pathogenesis of the chronic disease and revealed the role of DII in incidence of cardiovascular disease [20], lung function [21], bone health [22] and some types of cancers [23, 24]. However, no study is available evaluating the association of dietary inflammatory index with cardiovascular risk factors in patients candidate for CABG. The hypothesis of the current study is that higher DII scores (or higher DII quartiles) will be associated with higher values of CVD risk indicators.

## Methods

### Subjects

Participants in the current cross-sectional study were candidates for isolated CABG with cardiopulmonary bypass and were recruited for Tehran Heart Center-Coronary Outcome Measurement (THC-COM) study. The study was carried out between May-September 2006. Participants in this study were patients admitted to the cardiothoracic ward for CABG surgery at a large Heart Center in this time period (Tehran Heart Center, Iran). The sample size calculation has been explained before [25]; briefly, the sample size was calculated using the formula for comparing two proportions: n = [(Zα/2 + Zβ)2 × {(p_1_ (1-p_1_) + (p_2_ (1-p_2_))}]/ (p_1_ - p_2_)^2^ where p_1_ is the proportion of the women with low quality Mediterranean regimen (0.3), p_2_ is the proportion of the men with low quality Mediterranean regimen (0.25), α-error = 0.05, and power = 80% (1-β). Accordingly, a 125-subject sample size was determined for the study (125 in each group). We also assumed 20% loss (125 + 25) and as men with CAD are twice as women (150 + 300), the final sample size of 450 was considered for the study [25–27]. Reasons for drop-out or exclusion were incomplete dietary questionnaires (*n* = 1), and incomplete demographic questionnaires (*n* = 5). The final analytic sample in this study consisted of 454 patients aged 35-80 years who completed both the questionnaire and the medical examination. More details of study procedure and biochemical assays have been provided elsewhere [25]. Written informed consent was obtained from each participating subject. The study was approved by the Ethics Committee of Tehran Heart Center, Tehran University of Medical Sciences.

### Clinical assessment of patients

Clinical assessment and pre-operative cardiac status was also measured by several variables including: left ventricular ejection- fraction, number of diseased vessels, New York Heart Association (NYHA) functional class and the European system for cardiac operative risk evaluation (EuroSCORE) [28]. EuroSCORE is a simple, additive risk model of perioperative mortality and as a useful predictor of the long term hazard of cardiovascular events leading to death or hospital admissions after cardiac surgery [29]. It is calculated according to the standard additive methods and was assessed as a continuous variable [30]. NYHA functional classification provides a simple way of classifying the extent of heart failure. It places patients in one of four categories based on how much they are limited during physical activity; the limitations/ symptoms are in regard to normal breathing and varying degrees in shortness of breath and/or angina; NYHA classes are composed from four classes included from no symptoms, mild symptoms, marked limitation in activity due to symptoms and severe symptoms [31].

### Anthropometric assessments

Anthropometric variables including weight and height were measured and body mass index (BMI) was calculated. Weight was measured while subjects wearing light clothes [32].

### Dietary assessments and DII calculation

DII was calculated based on a 138-item semi-quantitative food frequency questionnaire (FFQ) consisting of a list of foods with standard serving sizes commonly consumed by Iranians. Participants were asked to report how often they consumed each of the food items listed as the number of times per day, per week, per month or per year during the previous year. The reported frequency for each food item was then converted to a daily intake. Portion sizes of consumed foods were converted to grams by using household measures [33]. The questionnaire was previously validated for healthy Iranian population [34].

DII is a population based index representing the pro-inflammatory or anti-inflammatory potential of a diet based on a scoring algorithm extracted form an extensive literature review while it has been explained in detailed elsewhere [17]. Briefly, dietary parameters are scored according to whether they had a pro-inflammatory effect (+ 1), anti-inflammatory (− 1) or no effect (0) based on six inflammatory biomarkers: IL-1β, IL-4, IL-6, IL-10, TNF-α and CRP. For calculating the DII, the dietary data were first linked to the world data base which provided a robust estimate of the mean and SD for each food variable considered [17]. Then, world means were subtracted from the actual intakes and divided by its standard deviation, creating a z score. To minimize the effects of right skewing, the z scores were converted into percentile scores. The centralized percentile scores of each food variable for each individual which was achieved by doubling the percentiles and subtracting 1 was multiplied by the respective effect score of food variable (inflammatory potential for each food variable). In the final step, all of the achieved values were summed across all food parameters to provide the total DII score. The greater the DII score, the more pro-inflammatory the diet. More negative values represent the more anti-inflammatory diets. The DII scores in the current research ranged from − 19.33 (maximally anti-inflammatory) to 10.62 (maximally pro-inflammatory). The DII computed based on this study’s FFQ included data on 28 of the 45 possible food variables composing the DII: energy, carbohydrate, protein, fat, fiber, cholesterol, poly unsaturated fatty acid (PUFAs), mono unsaturated fatty acid (MUFAs), omega-3, omega-6, *trans* fat, saturated fat, thiamin, riboflavin, niacin, vitamin B6, vitamin B12, folic acid, iron, zinc, magnesium, selenium, vitamin A, vitamin C, vitamin E, vitamin D, β-carotene, garlic, tea and caffeine. The food components were selected according to previously published articles regarding the most important relations of these food ingredients with cardiovascular risk factors [35]. To examine the relationship between DII scores and outcomes of interest, the DII was divided into quartiles with the following cut-points: Q1: − 29.83 to ≤ − 15.05, Q2: -15.04 to ≤ − 5.36, Q3: -5.35 to ≤ − 0.2 and Q4: − 0.19 to ≤7.01.

### Statistical analyses

Analysis of data was performed by SPSS software (statistical package for social analysis, version 18, SPSS Inc., Chicago, IL, USA). The normality of data was tested by Kolmogorov-Smirnov test and all parameters were normally distributed. The comparison of discrete and continuous variables between different quartiles of DII score was performed by Chi- square test and analysis of variance (ANOVA) respectively. Beta estimates and 95% confidence intervals (CI) for the association between different DII quartiles and clinical parameters such as HbA1c, HDL, LDL, TG, HCT and etc. were estimated using linear regression adjusting for confounders including age, gender, BMI, educational attainment and presence of diabetes and myocardial infarction. All data are expressed as means ± SD. A two-sided *P* value less than 0.05 was considered significant.

## Results

General demographic and anthropometric variables among patients according to DII quartiles are presented in Table 1. Patients in the top quartile of DII with more pro-inflammatory diet, had significantly higher age compared with patients in lower quartiles (*P* = 0.022). Gender distribution was also in favor of male in lower quartiles (*P* = 0.039). BMI was lower in top quartile compared with lowest quartile and patients in the highest DII quartile with the more inflammatory diet had significantly higher prevalence of diabetes and MI and had lower educational attainment (*P* < 0.05). Tables 2 and 3 presents the β estimate and confidence interval (CI) for the association between DII and biochemical variables in male and female patients candidate for CABG respectively; male patients in 4th quartiles of DII had significantly higher TC, TG, Albumin, creatinine and BUN concentrations and lower HDL concentrations compared with male patients in 3rd quartile (*P* < 0.05). Also, male patients in 3rd quartile had significantly higher HbA_1_C, TC and CRP and lower HDL concentrations compared with male patients in 2nd quartile (P < 0.05). Finally, male patients in 2nd quartile had significantly higher TG concentrations compared with patients in reference quartile.

**Table 1:** General demographic and anthropometric parameters in patients candidate for CABG

Quartiles of DII score					
Variable	1^st^ quartile	2^nd^ quartile	3^rd^ quartile	4^th^ quartile	*P* value
	*N* = 113	*N* = 113	*N* = 113	*N* = 113	
Age (y)	57.94 ± 8.58	59.89 ± 9.07	57.51 ± 8.80	60.67 ± 9.23	0.022
Gender male [n (%)]	89 (78.8)	84 (74.3)	83 (73.5)	76 (67.3)	0.039
BMI (kg/m^2^)	27.25 ± 3.75	27.25 ± 3.75	27.73 ± 3.90	26.49 ± 3.92	0.009
Diabetic [n (%)]	43 (38.1)	49 (43.3)	47 (41.6)	52 (46)	0.048
High education level [n (%)]	17 (15.3)	18 (16.2)	17 (15.5)	14 (13)	0.049
Smokers [n (%)]	45 (39.8)	42 (37.2)	40 (35.7)	31 (27.4)	0.054
Hyperlipidemia [n (%)]	77 (68.1)	78 (69)	87 (77)	79 (69.9)	0.48
Hypertension [n (%)]	59 (52.2)	57 (50.4)	45 (40.2)	55 (48.7)	0.32
MI [n (%)]	50 (45)	55 (49.1)	60 (53.1)	61 (54)	0.029

*BMI* body mass index, *MI* myocardial Infarction. P value for discrete variables based on Chi-Square Test and for continuous variables based on ANOVA. Discrete and continuous variables data are presented as number (percent) and mean (SD). High educational attainment was defined as educational level more than 12 years

**Table 2:** β estimate and confidence interval (CI) for the association between DII and biochemical variables in male patients candidate for CABG

Quartiles of DII score				
Variable	1^st^ quartile	2^nd^ quartile	3^rd^ quartile	4^th^ quartile
	*N* = 89	*N* = 84	*N* = 83	*N* = 76
HbA1C (%)	1 (Ref.)	1.03 (0.85-1.24)	1.18 (0.99-1.41)*	0.89 (0.71 – 1.12)
TC (mg/dl)	1 (Ref.)	1.02 (0.99-1.04)	1.01 (1.00-1.03)*	1.02 (0.99-1.04)*
TG (mg/dl)	1 (Ref.)	0.99 (0.98 – 1.00)*	0.99 (0.99-1.002)	0.99 (0.98 -0.99)*
LDL (mg/dl)	1 (Ref.)	0.98 (0.96-1.02)	0.99 (0.98-1.01)	1.001 (0.98-1.02)
HDL (mg/dl)	1 (Ref.)	0.96 (0.92-1.01)	−0.95 (0.90-0.99)*	−0.95 (0.91-1.00)*
HCT (%)	1 (Ref.)	0.99 (0.93-1.05)	1.02 (0.96-1.07)	0.99 (0.93-1.06)
Albumin (g/dL)	1 (Ref.)	0.64 (0.25-1.68)	0.61 (0.23-1.63)	0.41 (0.14-1.14)*
Creatinine (mg/dL)	1 (Ref.)	1.31 (0.32-5.39)	0.84 (0.17-3.96)	0.14 (0.02-0.82)*
BUN (mg/dL)	1 (Ref.)	1.02 (0.99-1.05)	1.009 (0.97-1.04)	1.04 (1.01-1.08)*
Lipoprotein (a) (mg/dL)	1 (Ref.)	1.004 (0.99-1.01)	1.01 (0.99-1.02)	1.01 (0.99 – 1.02)
CRP (mg/dL)	1 (Ref.)	0.96 (0.89-1.05)	0.87 (0.76 -0.99)*	0.97 (0.89-1.06)

*Hb* hemoglobin, *TC* total cholesterol, *TG* triglyceride, *LDL* low density lipoprotein cholesterol, *HDL* high density lipoprotein cholesterol, *HCT* hematocrit, *BUN* blood urea nitrogen, *CRP* C-reactive protein. The β estimate and confidence interval (CI) was estimated using linear regression model adjusting for the confounding effects of age, gender, BMI, educational attainment and presence of diabetes and myocardial infarction.* Indicates statistically significant values as *P* < 0.05

**Table 3:** β estimate and confidence interval (CI) for the association between DII and biochemical variables in female patients candidate for CABG

Quartiles of DII score				
Variable	1^st^ quartile	2^nd^ quartile	3^rd^ quartile	4^th^ quartile
	*N* = 22	*N* = 22	*N* = 29	*N* = 36
HbA1C (%)	1 (Ref.)	0.77 (0.51-1.17)	0.89 (0.59-1.34)	0.88 (0.59 – 1.31)
TC (mg/dl)	1 (Ref.)	0.99 (0.97-1.01)	0.74 (0.37-1.48)	0.67 (0.34-1.37)
TG (mg/dl)	1 (Ref.)	1.01 (0.99-1.02)	1.05 (0.92-1.21)	1.08 (0.94-1.25)
LDL (mg/dl)	1 (Ref.)	0.99 (0.96-1.02)	1.32 (0.66-2.63)	1.46 (0.72-2.97)
HDL (mg/dl)	1 (Ref.)	1.01 (0.94-1.09)	1.29 (0.65-2.57)	1.42 (0.7-2.88)
HCT (%)	1 (Ref.)	1.25 (1.00-1.56)*	1.18 (0.94-1.47)	1.08 (0.88-1.34)
Albumin (g/dL)	1 (Ref.)	1.61 (0.22-5.87)	1.48 (0.21-5.12)	2.25 (0.34-10.11)
Creatinine (mg/dL)	1 (Ref.)	7.56 (0.1-52.30)	1.79 (0.02-12.9)	2.03 (0.03-10.11)
BUN (mg/dL)	1 (Ref.)	1.00 (0.94-1.07)	0.99 (0.92-1.06)	1.02 (0.95-1.08)
Lipoprotein (a) (mg/dL)	1 (Ref.)	0.99(0.97 -1.01)	0.98 (0.96-1.00)	0.98 (0.96-1.00)*
CRP (mg/dL)	1 (Ref.)	1.07 (0.8-1.43)	1.24 (0.96-1.61)	0.93 (0.67-1.30)

*Hb* hemoglobin, *TC* total cholesterol, *TG* triglyceride, *LDL* low density lipoprotein cholesterol, *HDL* high density lipoprotein cholesterol, *HCT* hematocrit, *BUN* blood urea nitrogen, *CRP* C-reactive protein. The β estimate and confidence interval (CI) was estimated using Linear regression model adjusting for the confounding effects of age, gender, BMI, educational attainment and presence of diabetes and myocardial infarction.* Indicates statistically significant values as *P* < 0.05

**Fig. 1:**
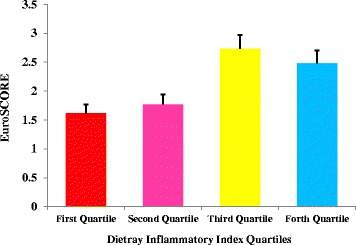
European system for cardiac operative risk evaluation (EuroSCORE) in patients according to DII quartiles; significant difference between 3rd and 4th quartiles with first and second quartiles; (*P* = 0.006 and *P* = 0.001 respectively)

**Fig. 2:**
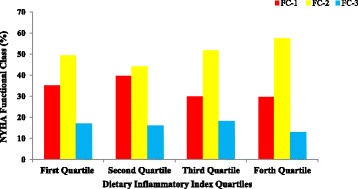
New York Heart Association (NYHA) functional class in patients according to DII quartiles; no significant difference between different quartiles of DII has been observed

**Fig. 3:**
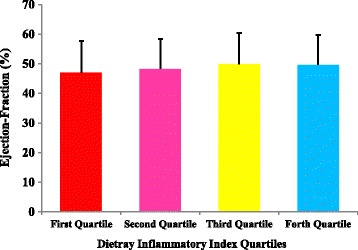
Left ventricular ejection-fraction in patients according to DII quartiles; no significant difference between different quartiles of DII has been observed

**Fig. 4:**
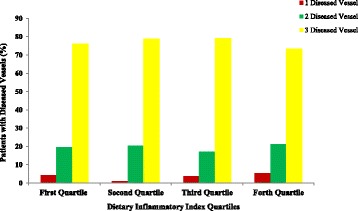
Number of diseased vessels in patients according to DII quartiles; no significant difference between different quartiles of DII has been observed

In female patients, only lipoprotein (a) concentrations and hematocrit (HCT) percentage in the 4th and 2nd quartile were significantly higher than lower quartiles. There was no significant association between other parameters and DII among participants. Figures 1, 2, 3 and 4 presents the pre-operative cardiac status including EuroSCORE, NYHA functional class, left ventricular ejection-fraction rate and number of diseased vessels among patients. As shown in these figures, only EuroSCORE was significantly higher in 3rd and 4th quartiles compared with first and second quartiles (*P* = 0.006, *P* = 0.001 respectively). Other clinical parameters were similarly distributed between different quartiles.

## Discussion

According to the results of the current work, patients in the higher DII scores had significantly higher age, higher serum lipids, HbA_1_C, albumin, creatinine, BUN, CRP and hematocrit (HCT) compared with patients with lower DII scores. Moreover, these patients had significantly higher prevalence of diabetes, myocardial infarction and lower educational attainment.

Inflammation plays an important role in the pathogenesis of numerous chronic diseases; in other word, inflammation is a leading cause of CVD, diabetes, obesity and almost all types of cancers and also play an important role in the progression of these disease to their advanced from [36]. Among them, inflammation is a well-known cause of cardiovascular events; indeed, atherosclerosis is an inflammatory process; numerous inflammatory molecules including hs-CRP, IL-6, IL-1β and TNF-α are elevated in patients with myocardial infarction or unstable angina [37, 38]. Several studies have reported that anti-inflammatory agents are potent reducers of cardiovascular events [39, 40].

Limited amounts of previous studies reported the prognostic value of DII in determination of the risk of several chronic diseases. In the study by Ramallal et al. [41] and the study by Garcia- Calzon et al. [35] the association between DII and the incidence of cardiovascular events has been reported. Also, its relation with metabolic parameters like higher CRP concentrations [16] or markers of glucose intolerance [42] and insulin resistance [43] has been confirmed in several previous studies. However, its association with the cardiovascular risk factors in patients candidate for CABG has not been evaluated before. Numerous dietary quality indices have been developed before and they are typically based on priori dietary guidelines definitions. Among them, healthy eating index (HEI), modified healthy eating index (MHEI), dietary approach to stop hypertension dietary pattern (DASH) and dietary diversity score (DDS) has been developed and their association with chronic disease has been revealed [44–46]. However, dietary inflammatory index, as a newly developed literature review based inflammatory index, has an additional benefit in measuring the dietary inflammatory potential, as an important risk factor of numerous disease especially cardiovascular disease [47]. DII is also in a strong agreement with other dietary quality indices and its additional advantage of measuring the inflammatory potential of a diet, makes it as a useful tool for determination of the association between diet and inflammatory disease [47].

The positive association of the DII with serum TC, TG and negative association with HDL in male patients is not out of expects. Inflammation is a potent trigger of dyslipidemia and leads to change in lipid metabolism. Acute phase response, mediated by pro-inflammatory cytokines like CRP, IL-6 and IL-8, in its chronic state, leads to chronic disorders including atherosclerosis and cardiovascular events. The activation of the inflammatory cascade leads to reduced HDL-cholesterol (HDL-C), with impairment in reverse cholesterol transport, and parallel changes in apo-lipoproteins, enzymes, anti-oxidant capacity and adenosine three-phosphate (ATP) binding cassette A_1_-dependent efflux. This decrease in HDL-C and phospholipids could stimulate compensatory changes, as synthesis and accumulation of phospholipid-rich very low density lipoprotein cholesterol (VLDL-C) which binds bacterial products and other toxic substances, resulting in hypertriglyceridemia. The final consequence is an increased accumulation of cholesterol in cells [48, 49].

Accordingly, higher prevalence of diabetes and HbA1C concentrations in patient with higher DII scores could be explained by this fact that type 2 diabetes is strongly associated with increased inflammation. Increased inflammation in adipose tissue is a strong driving force for the development of increased systemic inflammation that results in type 2 diabetes [50]. According to previous reports, the potential reversal of diabetes can be achieved by reducing the levels of inflammation through the use of an anti-inflammatory diet. It has been proposed that high-inflammatory diet characterized by *(a)* increased consumption of refined high-glycemic load carbohydrates, *(b)* increased consumption of refined vegetable oils rich in omega-6 fatty acids, and *(c)* decreased consumption of long-chain omega-3 fatty acids leads to entry of a diet with high glycemic load into the blood stream and high production of insulin is responsible for silent inflammation in diabetes [51].

Accordingly, the positive association between serum creatinine and BUN concentrations with DII in male patients, was also a confirmation of previously published works reporting the role of inflammatory diet in promoting kidney disease and lower kidney function [52]. In the study by Xu et al. the higher adapted dietary inflammatory index (ADII) was associated with systemic inflammation and reduced kidney function demonstrated by higher CRP and lower glomerular filtration rate (GFR) in elderly adults [52]. Also, in another study by Kizil et al. [53], dietary inflammatory index was associated with higher CRP and slightly higher creatinine concentrations in patients with hemodialysis.

In our study, the BMI in the second quartile of DII was higher than the forth quartile (*P* = 0.009). This contradictory finding was also in agreement of the findings of the Kizil et al. study [53] reporting a negative association between DII and BMI. Similarly, in the study by Zamora-Ros et al. [54], the BMI in the second quartile of DII was higher than forth quartile among patients with colorectal cancer. In fact it has been confirmed that BMI does not assess central adiposity associated with cardiovascular disease and inflammation and central obesity indices are more important CVD predictors compared with BMI [55]. Likewise, in the current study and also in the Kizil study, BMI failed to assess inflammatory state of participants. Similarly, it was reported that the severity of CVD and mortality was not related to BMI even in normal, overweight, and obese patients [56]. Therefore, it could be suggested that BMI lacks the discriminatory power to differentiate between body fat and lean mass among CVD patients.

Another finding, the gender difference in the association between DII and biochemical variables should be discussed here; as mentioned in the results, while in the male participants the DII was in negative association with serum lipids, CRP, BUN and creatinine as discussed above, in females, only HCT and lipoprotein (a) were positive predictors of DII. The incidence of cardiovascular disease is known to be higher in men than in women of similar age, and this gender difference is more prominent at a younger age [57] and is partly explained by protective effects of sex hormones [58]. On the other hand, female patients with CAD have been reported to be more likely to have a worse cardiovascular risk factor profile [59].

Other interesting finding of the current report was the higher EuroSCORE in higher DII categorizes. However, the number of diseased vessels, NYHA functional class and left ventricular ejection fraction were similar between different DII categorizes. The EuroSCORE predictive tool is applied in cardiac surgeries in adult patients to predict the cardiovascular mortality with good or excellent predictive ability [60, 61]. In one study by Lomivorotov et al. [62] nutritional status evaluated by nutritional screening questionnaires was associated with logistic EuroSCORE (odd’s ratio = 1.06, 95% CI:1-1.1). However, no study evaluated the association of EuroSCORE with DII in these patients.

Several limitations of the current study should also be addressed; the self-reported dietary information achieved by FFQ could address a potential recall bias. However, the validity and reliability of the questionnaire has been confirmed before. Secondly, the central adiposity indices like WHR or WC as better predictors of inflammation in CVD were not measured in the current study. However, the relatively large sample size, inclusion of multiple covariates in the statistical model and using numerous clinical indicators of CVD like EuroSCORE and NYHA classification scores are potent strengths of the current study. Moreover, this is the first study evaluated the association between DII and CVD risk factors in patients candidate for CABG. The importance of dietary interventions, healthy dietary habits and the urgent need to nutrition education for patients undergoing CABG for prevention of recurrent cardiac events further highlights the clinical applications of the current findings.

## Conclusion

DII has strong association with cardiovascular risk factors in patients candidate for CABG. These associations were gender-specific with more pronunciation in men. Further studies are needed to evaluate the potential use of DII as a global measure of inflammatory potential of diet in relation to cardiovascular risk factors in patients candidate for CABG.

## Abbreviations

ANOVA: Analysis of variance
CABG: Coronary artery bypass grafting surgery
CAD: Coronary artery disease
CRP: C-reactive protein
CVD: Cardiovascular disease
DII: Dietary inflammatory index
EuroSCORE: Functional class and the European system for cardiac operative risk evaluation
HDL: High density lipoprotein cholesterol
IL: Interleukin
LDL: Low density lipoprotein cholesterol
LP: Lipoprotein
NYHA: New York Heart Association
TC: Total cholesterol
TNF-α: Tumor necrosis factor- α
TNF-α: Tumor necrosis factor-α
